# Arterial pulse wave propagation across stenoses and aneurysms: assessment of one-dimensional simulations against three-dimensional simulations and *in vitro* measurements

**DOI:** 10.1098/rsif.2020.0881

**Published:** 2021-04-14

**Authors:** Weiwei Jin, Jordi Alastruey

**Affiliations:** ^1^ Department of Biomedical Engineering, King’s College London, London, UK; ^2^ World-Class Research Center ‘Digital Biodesign and Personalized Healthcare’, Sechenov University, Moscow, Russia

**Keywords:** haemodynamics, mathematical modelling, pulse wave propagation, computational fluid dynamics, *in vitro* measurements

## Abstract

One-dimensional (1-D) arterial blood flow modelling was tested in a series of idealized vascular geometries representing the abdominal aorta, common carotid and iliac arteries with different sizes of stenoses and/or aneurysms. Three-dimensional (3-D) modelling and *in vitro* measurements were used as ground truth to assess the accuracy of 1-D model pressure and flow waves. The 1-D and 3-D formulations shared identical boundary conditions and had equivalent vascular geometries and material properties. The parameters of an experimental set-up of the abdominal aorta for different aneurysm sizes were matched in corresponding 1-D models. Results show the ability of 1-D modelling to capture the main features of pressure and flow waves, pressure drop across the stenoses and energy dissipation across aneurysms observed in the 3-D and experimental models. Under physiological Reynolds numbers (*Re*), root mean square errors were smaller than 5.4% for pressure and 7.3% for the flow, for stenosis and aneurysm sizes of up to 85% and 400%, respectively. Relative errors increased with the increasing stenosis and aneurysm size, aneurysm length and *Re*, and decreasing stenosis length. All data generated in this study are freely available and provide a valuable resource for future research.

## 1. Introduction

Computational blood flow modelling can provide valuable insights into the assessment of cardiovascular disease. The most common formulations for vascular blood flow modelling are zero-dimensional (0-D), one-dimensional (1-D) and three-dimensional (3-D) models. Lumped parameter (or 0-D) models provide compartmental representations of the cardiovascular system. They have been used to study whole-body haemodynamics, including cardiac dynamics [1], and time-variant haemodynamics involving activities such as postural change [2] and exercise [3,4]. A 0-D approach is computationally inexpensive (analytical solutions are often possible), but is not suitable for describing pulse wave propagation and complex flow phenomena occurring in blood vessels. On the other hand, 3-D modelling based on the incompressible Navier–Stokes equations can describe pulse wave propagation [5] and complex flow phenomena; for example, 3-D flow patterns and mechanical stresses resulting from interactions between blood flow and the vessel wall in stenoses [6,7] and aneurysms [8,9]. However, this comes at a considerably higher computational cost than the 0-D and 1-D modelling approaches [10,11]. 3-D simulations require of the order of hours (even days) to compute the solution using high-performance computers, whereas 0-D and 1-D models take of the order of seconds or minutes on standard PCs.

Several studies have shown that 1-D models—in which the 3-D space dependency is reduced to the vessel’s axial coordinate only—are a good compromise between accuracy and computational cost to simulate pulse wave propagation in large arterial networks. This has been achieved through various comparisons of 1-D model pressure, flow and cross-sectional area waves against *in vitro* data acquired in experimental set-ups of the aorta and its larger branches made of flexible tubes [12–15], *in vivo* data acquired in humans [16–19] and animals [20,21], and numerical data obtained by solving the full 3-D equations of blood flow in compliant vessels [11,15,19]. These studies have mainly focused on assessing the accuracy of 1-D modelling under normal anatomical conditions, which are characterized by the presence of tapering, junctions and vessel curvature and torsion. They reported relative root mean square errors (RMSEs) between 1-D model and reference waveforms of as little as 1.2% for pressure, 2.1% for the flow and 2.6% for the cross-sectional area (table 1).

**Table 1.** Literature review of studies on 1-D modelling accuracy. The third column shows the type of reference data used in each study. Upper bounds for relative errors for pressure (ɛ_*P*_), flow rate (ɛ_*Q*_), flow velocity (ɛ_*U*_) and cross-sectional area (ɛ_*A*_) wave morphology, calculated as described in the corresponding article, are shown when available.

reference		test data	simulated arteries	ɛ_*P*_	ɛ_*Q*_	ɛ_*U*_	ɛ_*A*_
Matthys *et al.*	[12]	*in vitro*	37 larger arteries	4.0%	19.0%	—	—
Bessems *et al.*	[22]	*in vitro*	Ao^a^	*	*	—	—
Alastruey *et al.*	[13]	*in vitro*	37 larger arteries	2.5%	10.8%	—	—
Saito *et al.*	[14]	*in vitro*	9 larger arteries	10.0%	*	—	—
Huberts *et al.*	[23]	*in vitro*	upper-limb arteries	*	*	—	—
Boileau *et al.*	[15]	*in vitro*	37 larger arteries	4.0%	25.6%	—	—
Avolio	[24]	human	128 larger arteries	—	—	*	—
Stettler *et al.*	[25,26]	human	Ao and lower-limb arteries	*	*	—	—
Olufsen *et al.*	[27]	human	29 larger arteries	—	*	—	—
Reymond *et al.*	[16]	human	103 larger arteries	*	*	—	—
Reymond *et al.*	[17]	human	94 larger arteries	6.0%^b^	11.0%	—	—
Willemet *et al.*	[18]	human	lower-limb arteries	9.6%	—	16.0%	—
Alastruey *et al.*	[19]	human	upper Ao and supra Ao arteries	10.0%	7.0%	—	8.0%
Strocchi *et al.*	[28]	human	55 larger arteries	*	*	—	—
Steele *et al.*	[20]	animal	aortic bypass	—	4.2%		
Mynard *et al.*	[21]	animal	left conduit coronary arteries	—	16.7%	—	—
Mynard *et al.*	[29]	3-D data	carotid bifurcation	—	—	*	—
Grinberg *et al.*	[30]	3-D data	50 larger intracranial arteries	*	*	—	—
Xiao *et al.*	[11]	3-D data	CCA, thoracic Ao, aortic bifurcation	1.4%	2.1%	—	2.6%
Xiao *et al.*	[11]	3-D data	20 larger arteries	2.1%	4.9%	—	—
Boileau *et al.*	[15]	3-D data	CCA, thoracic Ao, aortic bifurcation	1.2%	2.6%	—	4.3%
Alastruey *et al.*	[19]	3-D data	upper Ao and supra Ao arteries	2.0%	5.0%	—	3.0%

Ao, aorta; CCA, common carotid artery.

*Qualitative comparison only.

— No comparison made.

^a^According to the dimensions shown in fig. 4 of [22].

^b^Except at the abdominal aorta, where root mean square error is 21%.

The effect on 1-D modelling accuracy of localized changes in vascular geometry—for example those occurring in diseased vasculatures with stenoses and aneurysms—has received less attention. Sterogiopulos *et al.* investigated the effects of arterial stenoses on arterial pulse waveforms, though the accuracy of the 1-D formulation used in their studies was evaluated by qualitative comparisons with previous computational and *in vivo* studies [31] and quantitative comparisons of stenosis reflection coefficients against *in vitro* data [32], without assessing 1-D model pressure and flow waveforms. Wan *et al.* [33] computed 1-D and 3-D model haemodynamics on a patient-specific arterial network of the abdominal aorta and larger lower-limb arteries with several localized changes in luminal cross-sectional area due to the presence of occlusive disease. However, they focused their study on assessing mean flow rates rather than pulse waveforms. In all those studies, pressure losses across stenoses were described in the 1-D formulation based on empirical data [34,35]. Papadakis & Raspaud [36] compared 1-D model pressure and flow velocity data obtained both analytically and computationally in an idealized stenotic carotid artery (with a 75% stenosis size) against corresponding data obtained using a 3-D model. They showed a very good agreement between 1-D and 3-D model flow waveforms, with relative errors smaller than 1.0%, though the 1-D model pressure wave was severely underpredicted at the inlet. A small number of studies have simulated blood flow across aneurysms using 1-D modelling [37–39]. These include comparisons against experimental measurements in idealized [38] and anatomically correct [39] geometries; yet the accuracy of 1-D model pulse waveforms was not quantified.

Geometrical multiscale models in which the 3-D, 1-D and 0-D formulations are mathematically coupled to form a unique model [40–42] have also been used to simulate blood flow in vessels with aneurysms and/or stenoses [43–46]. This alternative numerical approach reduces the computational cost of full 3-D models, though 3-D/1-D/0-D multiscale modelling is still computationally more expensive than 1-D modelling (coupled to 0-D outflow boundary conditions), which is the focus of this study.

**Figure 1.**
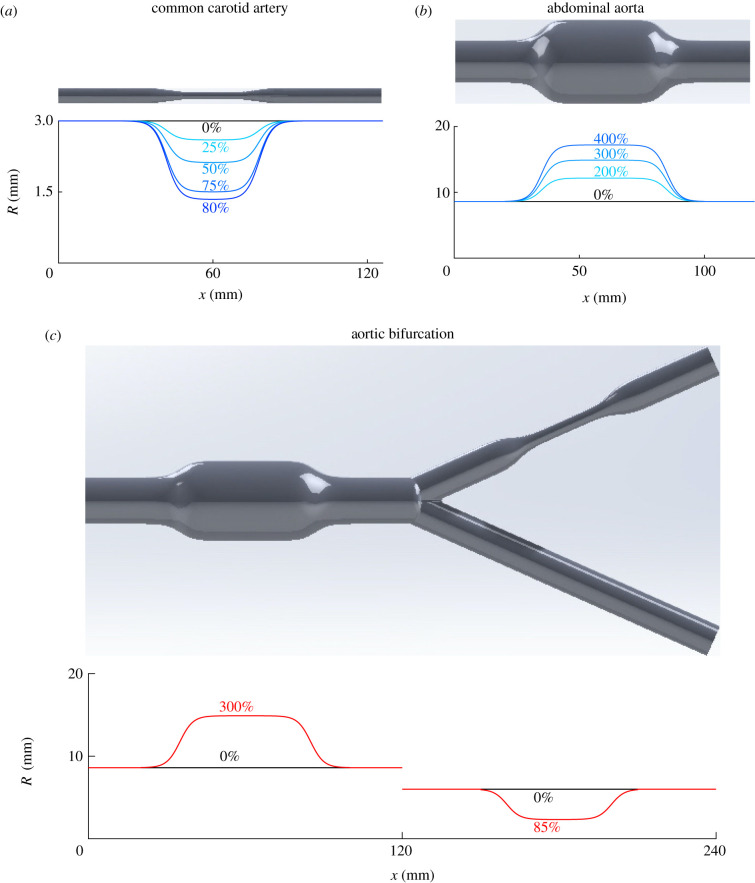
The idealized vascular geometries studied: common carotid artery with a stenosis (*a*), abdominal aorta with an aneurysm (*b*) and aortic bifurcation with an aneurysm in the abdominal aorta and a stenosis in the left iliac artery (*c*). Their luminal radius variations are shown in the corresponding plots, at baseline (black lines) and for different percentage degrees of stenosis and aneurysm (coloured lines).

**Figure 2.**
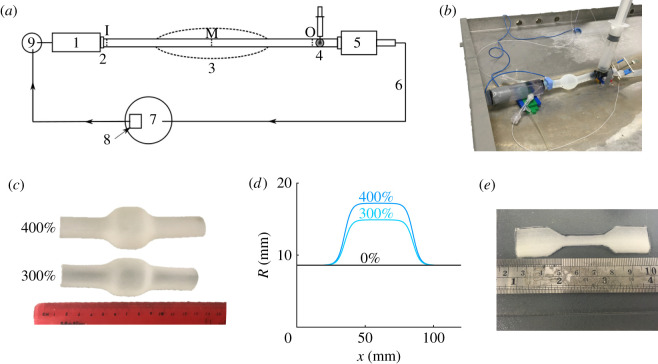
*In vitro* experimental set-up. (*a*) Schematic showing the piston-driven ventricle (1); aortic valve (2); aneurysm site (3); Windkessel model (4); peripheral drain box (5); guttering system (6); reservoir (7); submersible pump (8); and atrium (9). (*b*) Photo of the aortic part of the experimental set-up. (*c*) 300% and 400% aneurysm phantoms made of Agilus 30 clear resin. (*d*) Luminal radius variations. (*e*) Specimen for material property testing. The three perpendicular dash lines (I, inlet; M, middle; O, outlet) along the phantom in (*a*) indicate the sites where 1-D model pressures were compared with *in vitro* measurements.

This study aims to assess the ability of the 1-D formulation to simulate pulse wave propagation in arteries with localized changes in cross-sectional area due to the presence of a stenosis and/or aneurysm. 1-D model pressure and flow waveforms were compared against corresponding waveforms simulated by a 3-D formulation of blood flow in compliant vessels with identical boundary conditions and equivalent vascular geometries and material properties. We considered three idealized vascular geometries representing a human (i) common carotid artery (CCA) with a stenosis (figure 1*a*), (ii) abdominal aorta with an aneurysm (i.e. an AAA) (figure 1*b*), and (iii) aortic bifurcation with an AAA and a stenosis in the left iliac artery (figure 1*c*). Furthermore, 1-D model pressure waveforms in the abdominal aorta with an AAA were compared against *in vitro* measurements acquired in a 1 : 1 scale cardiovascular simulator rig of the human aorta (figure 2).

## 2. Methods

### 2.1. 1-D and 3-D simulations

#### 2.1.1. Generating vascular geometries

All 3-D vascular geometries used in this study were created using SolidWorks (Dassault Systémes SolidWorks Corporation, Vélizy-Villacoublay, France). They were obtained by rotating the radius variations shown in figure 1 about the centreline of the corresponding vessels. Vascular dimensions were based on the values provided by Xiao *et al.* [11]. A range of stenosis lengths and sizes representative of real human stenoses [47] were simulated in the middle of the CCA using the luminal radius variations shown in figure 1*a*. The size of the stenosis was defined as $(1-A_{S}/A_{0})\times 100\%$, with *A*_S_ the minimum luminal cross-sectional area within the stenosis and *A*_0_ the area proximal to the stenosis. A range of aneurysm lengths and sizes representative of real human aneurysms [48,49] were simulated in the middle of the abdominal aorta using the luminal radius variations shown in figure 1*b*. The size of the aneurysm was defined as $A_{A}/A_{0}\times 100\%$, with *A*_A_ the maximum luminal cross-sectional area within the aneurysm and *A*_0_ the area proximal to the aneurysm.

For each geometry, 3-D tetrahedral meshes were generated using MeshSim (Simmetrix Inc., NY, USA), with an absolute mesh size of 0.3 mm for the CCA and 0.45 mm for the abdominal aorta and aortic bifurcation. Boundary layer meshing of three layers was applied to all cases, with a total thickness of 0.5 mm for the CCA and 1 mm for the abdominal aorta and aortic bifurcation. The total number of elements in each case varied depending on the size of the stenosis or aneurysm; from 985 826 to 1 490 212 for the CCA, from 2 514 454 to 5 959 180 for the abdominal aorta and from 5 016 316 to 7 029 817 for the aortic bifurcation. The characteristics of the meshes used for the baseline geometries (i.e. without a stenosis or aneurysm) are based on the equivalent geometries in [11], for which mesh independence studies were undertaken. Compatible 1-D geometries with the same radius variation as the 3-D geometries were generated using Matlab (The MathWorks, Natick, MA, USA). Finite-element meshes with a size of up to 1.2 cm were used for all simulations.

#### 2.1.2. 1-D and 3-D haemodynamic models

**Figure 3.**
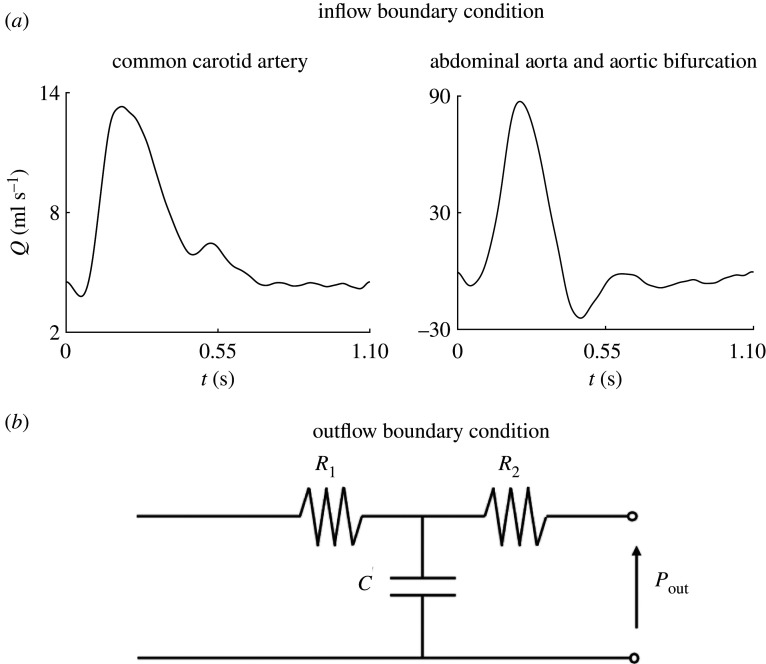
Inflow (*a*) and outflow (*b*) boundary conditions used in this study. The parameters of the outflow boundary conditions are shown in table 2.

The 1-D formulation was solved using our in-house solver Nektar1D [50]; 3-D simulations were performed using the open-source software CRIMSON [51]. Corresponding 1-D and 3-D simulations shared compatible geometries and material properties and had identical inflow/outflow boundary conditions. Fully developed Poiseuille flows (figure 3*a*) were prescribed at the inlets and three-element Windkessel models (figure 3*b* and table 2) were coupled at the outlets. An empirically based model [33] was used to calculate pressure losses across the stenosis in the 1-D simulations, hereafter referred to as the ‘stenosis model’. All 1-D simulations were run on a standard PC (MacBook Pro; 2.7 GHz Quad-Core Intel Core i7), whereas all 3-D model simulations were run on a high-performance computer (SGI Altix UV 1000). All 1-D and 3-D simulations were run for multiple cardiac cycles until a periodic solution was achieved in which the values of diastolic blood pressure at the start and end of the last cycle differed by less than 0.5%. The haemodynamic properties of the three models in figure 1 were taken from the properties of the corresponding models in Xiao *et al.* [11]. All models in this study considered blood to be an incompressible Newtonian fluid and blood flow to be laminar.

**Table 2.** Parameters of the outflow three-element Windkessel models (figure 3*b*) used in this study. Outflow blood pressures *P*_out_ were assumed to be zero in all cases.

	common carotid artery	abdominal aorta	aortic bifurcation
*R*_1_ (mmHg s ml^−1^)	1.8658	0.0881	0.5110
*C* (ml mmHg^−1^)	0.0234	1.3550	0.0489
*R*_2_ (mmHg s ml^−1^)	14.0239	0.8376	23.2617

#### 2.1.3. 1-D formulation

The governing equations of the 1-D model were [50]

2.1 $$\frac{\partial A}{\partial t}+\frac{\partial (AU)}{\partial x}=0,$$

2.2 $$\frac{\partial U}{\partial t}+U\frac{\partial U}{\partial x}+\frac{1}{\rho }\frac{\partial P}{\partial x}=\frac{\;f}{\rho A}$$

2.3 $$\;\mathrm{and}\;P=P_{d}+\frac{\beta }{A_{d}}\left(\sqrt{A}-\sqrt{A_{d}}\right),$$

where *A*(*x*, *t*) is the luminal cross-sectional area, *U*(*x*, *t*) is the blood flow velocity, *P*(*x*, *t*) is the arterial blood pressure, *t* is time and *x* is the axial coordinate. The density of blood, *ρ*, was taken to be 1060 kg m^−3^ to match the value used in the 3-D simulations. The frictional term per unit length, *f*(*x*, *t*) = *ηU*, was assumed to be proportional to *U*, with the viscous loss function, *η*, described in the next section. In the tube law (equation (2.3)), the subscript *d* denotes the diastolic value and *β* accounts for the stiffness of the arterial wall,

2.4 $$\beta =\frac{3}{4}\sqrt{\pi }Eh,$$

where *E* is Young’s modulus and *h* is the wall thickness. The time step for all 1-D simulations was 10^−5^ s.

#### 2.1.4. Stenosis model

An empirically based stenosis model [35] was introduced into the 1-D formulation through the frictional term *f* = *ηU* in equation (2.2), based on a previous study [33]. The viscous loss function, *η*, was the theoretical expression

2.5 $$\eta_{0}=-2(\zeta +2)\mu \pi \;$$

outside the stenosis and the empirical expression

2.6 $$\eta_{S}=\eta_{0}+\frac{-A_{S}^{2}Q_{0}^{2}\left[\frac{K_{v}}{Re_{0}}+\frac{K_{t}}{2}{\left(\frac{A_{0}}{A_{S}}-1\right)}^{2}\right]}{A_{0}^{2}Q_{S}L_{S}}\;$$

within the stenosis. The subscript ‘0’ refers to the arterial region proximal and distal to the stenosis, whereas the subscript ‘S’ refers to the arterial region within the stenosis. Blood viscosity, *μ* = 4 mPa s, matches the value used in 3-D simulations, *Q* = *AU* is the blood flow, *Re*_0_ = *ρU*_0_*D*_0_/*μ* is the Reynolds number, with *U*_0_ the flow velocity at a given time and *D*_0_ the cross-sectional diameter, *L*_S_ is the length of the stenosis, *K*_t_ = 1.52 and

2.7 $$K_{v}=32\frac{L_{S}}{D_{0}}{\left(\frac{A_{0}}{A_{S}}\right)}^{2}.$$

#### 2.1.5. 3-D formulation

CRIMSON enabled us to solve a 3-D fluid–structure interaction (FSI) problem in which blood flow was governed by the 3-D Navier–Stokes equations for an incompressible Newtonian fluid. The FSI problem was solved by using the ‘enhanced’ membrane formulation proposed by Figueroa *et al.* [51], in which vessel wall displacements are described as a function of blood velocity and pressure at the interface. In addition, external tissue support was used in all simulations to account for the tethering exertion and to eliminate spurious and non-physiological oscillations [52]. A detailed description of the 3-D formulation can be found in [51]. The time step for all 3-D simulations was 2 × 10^−4^ s.

### 2.2. *In vitro* experimental set-up

**Figure 4.**
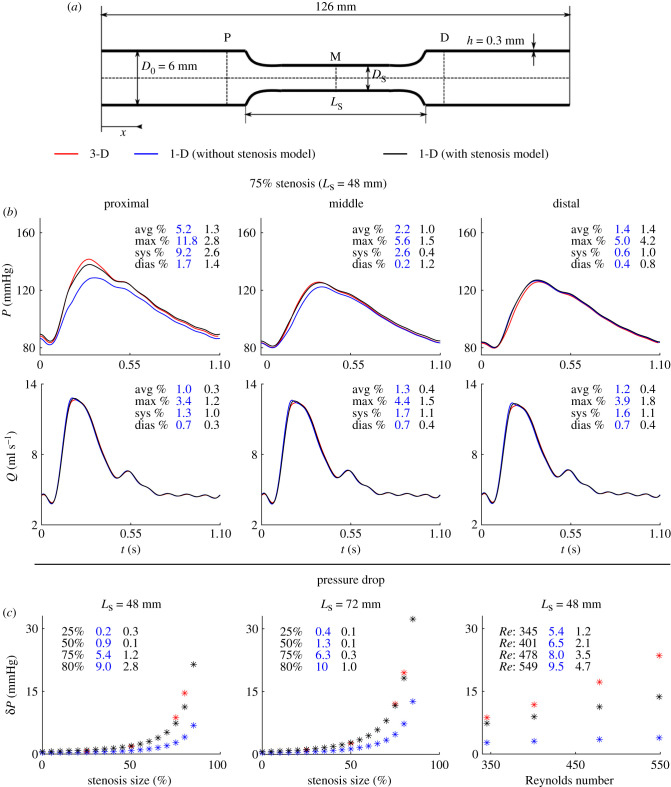
Results for the common carotid artery with a stenosis (1-D versus 3-D modelling). (*a*) The vascular geometry is characterized by the length of the stenosis, *L*_s_, the initial, *D*_0_, and stenosis, *D*_s_, diameters and the wall thickness, *h*. (*b*) Pressure and flow rate with time at the proximal (P), middle (M) and distal (D) sides shown in panel (*a*), calculated using the 3-D model (red) and 1-D model, with (black) and without (blue) the stenosis model, for a 75%, 48-mm-long stenosis with a Reynolds number of 345. Average (avg), maximum (max), systolic (sys) and diastolic (dias) relative error metrics are shown in each panel (column 1: without stenosis model; column 2: with stenosis model). (c) Pressure drop, *δP*, from the proximal to distal side with stenosis size, for a 48-mm-long stenosis (left) and a 72-mm-long stenosis (middle), and with Reynolds number (right) for a 75%, 48-mm-long stenosis (right), for the 3-D model (red stars) and 1-D model with (black stars) and without (blue stars) the stenosis model. 3-D model results shown for a few cases only. Relative errors *ε*_*δP*_ calculated by equation (2.9) with (column 1) and without (column 2) the stenosis model are shown in each panel.

The experimental cardiovascular simulator rig developed by Gaddum *et al.* [53] (figure 2*a*,*b*) was used to produce additional pulse wave data for the abdominal aorta case study with a 0%, a 300% and a 400% aneurysm (figure 2*c*,*d*). All AAA phantoms were made of Agilus 30 clear resin (Deed3d Technology Co., Ltd, Guangzhou, China) (samples shown in figure 2*c*). These flexible phantoms were fixed on the connectors after the aortic valve (figure 2*a*,(2)) and before the Windkessel model (figure 2*a*,(4)) in the experimental system using cable ties. Although some studies [54] have used blood-mimicking fluids, here water was used. As shown in figure 2*b*, an ultrasound-based flow sensor (COnfidence Flowprobes for Research (PAU Series); Transonic, Ithaca, NY, USA) and a pressure catheter (Mikro-Cath^™^ 3.5F; Millar, Houston, TX, USA) were fixed at the inlet of the abdominal aorta phantom to measure the flow and pressure waves. A second pressure catheter was used to measure the pressure wave at the inlet, middle and outlet of the aortic phantom. All flow and pressure sensors were calibrated at the start of each phantom measurement. The sampling rate of pressure and flow measurements was 1000 kHz. On average, 30 cardiac cycles were acquired at each measurement point. Samples from the phantom (figure 2*e*) were taken to estimate Young’s modulus using a uniaxial extension test. The test was performed using an electromechanical test system (5500 series; Instron, Wycombe, UK) on five different specimens taken from the AAA phantoms.

The data analysis was performed offline in Matlab. The signals were first filtered with a low-pass filter to reduce noise while maintaining the relevant frequency information. Subsequently, 15 beats were selected from the recorded beats and ensemble-averaged to generate a single beat pressure and flow waveform (with an ensemble standard deviation) to be compared with their numerical counterparts. The ensemble-averaged flow waves measured at the inlet of the aortic phantom were taken as the inflow boundary conditions for the 1-D simulations. The ensemble-averaged pressure measured by the first sensor fixed at the inlet of the aortic phantom was taken as a reference to calibrate the pressure measured by the second sensor at different positions along the phantom. Finally, the 1-D model parameters of the three-element Windkessel outflow boundary condition were calculated using the inflow boundary condition and the pressure waveform at the inlet using the algorithm described in [55].

### 2.3. Error calculations

The following relative error metrics were used to quantify the accuracy of the pressure, *P*, and flow, *Q*, waveforms computed by the 1-D formulation:

2.8 $$\begin{array}{cc} & \epsilon_{P}^{\mathrm{RMS}}=\sqrt{\frac{1}{n}\sum_{i=1}^{n}\left(\frac{P_{i}^{1D}-P_{i}^{\mathrm{Ref}}}{P_{i}^{\mathrm{Ref}}}\right)},\;\epsilon_{Q}^{\mathrm{RMS}}=\sqrt{\frac{1}{n}\sum_{i=1}^{n}\left(\frac{Q_{i}^{1D}-Q_{i}^{\mathrm{Ref}}}{\max(Q^{\mathrm{Ref}})}\right)},\\ & \epsilon_{P}^{\mathrm{MAX}}=\max_{i}|\frac{P_{i}^{1D}-P_{i}^{\mathrm{Ref}}}{P_{i}^{\mathrm{Ref}}}|,\;\epsilon_{Q}^{\mathrm{MAX}}=\max_{i}|\frac{Q_{i}^{1D}-Q_{i}^{\mathrm{Ref}}}{\max(Q^{\mathrm{Ref}})}|,\\ & \epsilon_{P}^{\mathrm{SYS}}=\frac{\max(P^{1D})-\max(P^{\mathrm{Ref}})}{\max(P^{\mathrm{Ref}})},\;\epsilon_{Q}^{\mathrm{SYS}}=\frac{\max(Q^{1D})-\max(Q^{\mathrm{Ref}})}{\max(Q^{\mathrm{Ref}})},\\ \; & \epsilon_{P}^{\mathrm{DIAS}}=\frac{\min(P^{1D})-\min(P^{\mathrm{Ref}})}{\min(P^{\mathrm{Ref}})},\;\epsilon_{Q}^{\mathrm{DIAS}}=\frac{\min(Q^{1D})-\min(Q^{\mathrm{Ref}})}{\max(Q^{\mathrm{Ref}})}, \end{array}\}$$

where *n* is the number of points in one cardiac cycle, $P_{i}^{1D}$ and $Q_{i}^{1D}$ are, respectively, the pressure and flow results obtained at each time point *i* = 1,…,*n* from the 1-D simulation at a single spatial location, and $P_{i}^{\mathrm{Ref}}$ and $Q_{i}^{\mathrm{Ref}}$ are the corresponding reference results obtained from the 3-D model or, for $P_{i}^{\mathrm{Ref}}$ only, the *in vitro* experimental set-up. 3-D model reference values were calculated as the cross-sectional averaged pressure and flow at each point *i* = 1,…,*n* at a single cross-section perpendicular to the vessel centreline. $\epsilon_{P}^{\mathrm{RMS}}$ and $\epsilon_{Q}^{\mathrm{RMS}}$ are the root mean square relative errors for pressure and flow; $\epsilon_{P}^{\mathrm{MAX}}$ and $\epsilon_{Q}^{\mathrm{MAX}}$ are the maximum relative errors for pressure and flow; $\epsilon_{P}^{\mathrm{SYS}}$ and $\epsilon_{Q}^{\mathrm{SYS}}$ are the relative errors in systolic pressure and flow; $\epsilon_{P}^{\mathrm{DIAS}}$ and $\epsilon_{Q}^{\mathrm{DIAS}}$ are the relative errors in diastolic pressure and flow. max(*Q*^Ref^) is the maximum value of the reference flow rate.

A relative error metric, *ε*_*δP*_ , was used to quantify the accuracy of the pressure drop (*δP*) estimated by the 1-D formulation (superscript ‘1D’) relative to the value obtained from the corresponding 3-D simulations (superscript ‘3D’),

2.9 $$\epsilon_{\delta P}=\frac{\left|\delta P^{1D}-\delta P^{3D}\right|}{{\bar{P}}_{\;\mathrm{prox}}^{3D}}.$$

*δP* was calculated as the difference in the mean blood pressure ($\bar{P}$) measured at 5 mm proximal and 5 mm distal to the stenosis. Notably, the denominator, ${\bar{P}}_{\;\mathrm{prox}}^{3D}$, is the mean blood pressure measured 5 mm proximal to the stenosis in the 3-D model.

In addition, the inaccuracies introduced by the inability of the 1-D formulation to simulate the intricate 3-D flow patterns within the aneurysm were assessed by comparing the energy dissipation between the 1-D and 3-D simulations. The energy dissipation, *E*_diss_, was calculated as the change in energy flux between the proximal (subscript ‘prox’) and distal (subscript ‘dist’) sites of the aneurysm [56],

2.10 $$\begin{array}{cc} & E_{\mathrm{diss}}=E_{\;\mathrm{prox}}-E_{\mathrm{dist}},\\ & E_{\;\mathrm{prox}}=Q_{\;\mathrm{prox}}\left(P_{\;\mathrm{prox}}+\frac{1}{2}\rho U_{\;\mathrm{prox}}^{2}\right)\\ \;\mathrm{and}\; & E_{\mathrm{dist}}=Q_{\mathrm{dist}}\left(P_{\mathrm{dist}}+\frac{1}{2}\rho U_{\mathrm{dist}}^{2}\right), \end{array}\}$$

where *E*_prox_ and *E*_dist_ are the energy flux at 5 mm proximal and 5 mm distal to the aneurysm, respectively. The following relative error metric ($\epsilon_{E_{\mathrm{diss}}}$) was used to quantify the accuracy of the energy dissipation estimated by the 1-D simulation (superscript ’1D’) relative to the value obtained from the 3-D simulation (superscript ‘3D’):

2.11 $$\epsilon_{E_{\mathrm{diss}}}=\frac{\left|E_{\mathrm{diss}}^{1D}-E_{\mathrm{diss}}^{3D}\right|}{E_{\;\mathrm{prox}}^{3D}}.$$

Notably, the denominator, $E_{\;\mathrm{prox}}^{3D}$, is the energy flux at 5 mm proximal to the aneurysm in the 3-D model.

## 3. Results

### 3.1. Assessment against 3-D model data

All idealized geometries representing typical CCA, AAA and aortic bifurcation with AAA and iliac stenosis shown in figure 1 were used for the systematic comparison of 1-D and 3-D modelling schemes. Each 1-D model simulation took less than 2 min for the CCA (18 cardiac cycles were run), 5 min for the abdominal aorta (30 cycles) and 9 min for the aortic bifurcation (30 cycles) on a standard PC, whereas corresponding 3-D model simulations required over 1 day for the CCA (10 cycles), 4 days for the abdominal aorta (12 cycles) and 7 days for the aortic bifurcation (16 cycles) using 64 processors on a high-performance computer. All relative errors for 1-D model waveforms obtained at baseline (i.e. without a stenosis or aneurysm) in the CCA, abdominal aorta and aortic bifurcation are consistent with the relative errors reported by Xiao *et al.* [11] in the same vascular geometries. These include root mean square relative errors (*ε*^RMS^) smaller than 1% for pressure and 2% for the flow for all cases. Electronic supplementary material, figures S1 (top), S6 (top) and S9 show comparisons of 1-D model pressure and flow waves against corresponding 3-D model waves in the CCA, abdominal aorta and aortic bifurcation, respectively, at baseline.

#### 3.1.1. CCA with a stenosis

Figure 4*b* compares 1-D model pressure and flow waveforms at 5 mm proximal, the middle and 5 mm distal to a 75%, 48-mm-long stenosis with corresponding 3-D model results. At those measurement sites, *ε*_RMS_ remained smaller than 1.5% for both pressure and flow waves when a stenosis of up to 75% in size was present in the middle of the vessel, for a physiological Reynolds number (*Re*) of 345. (All *Re* values reported in the Results section were calculated at peak flow velocity.) 1-D model pressure and flow waves were able to reproduce the main features of corresponding 3-D model waves, including the ripples observed for the pressure and flow waves in diastole. For all cases studied, all relative errors defined in §2.3 for pressure and flow waves were considerably smaller when the stenosis model was used, in particular for the pressure wave in sites proximal to the stenosis (e.g. ≤1.3% versus ≤5.2% for the 75%, 48-mm-long stenosis case).

**Figure 5.**
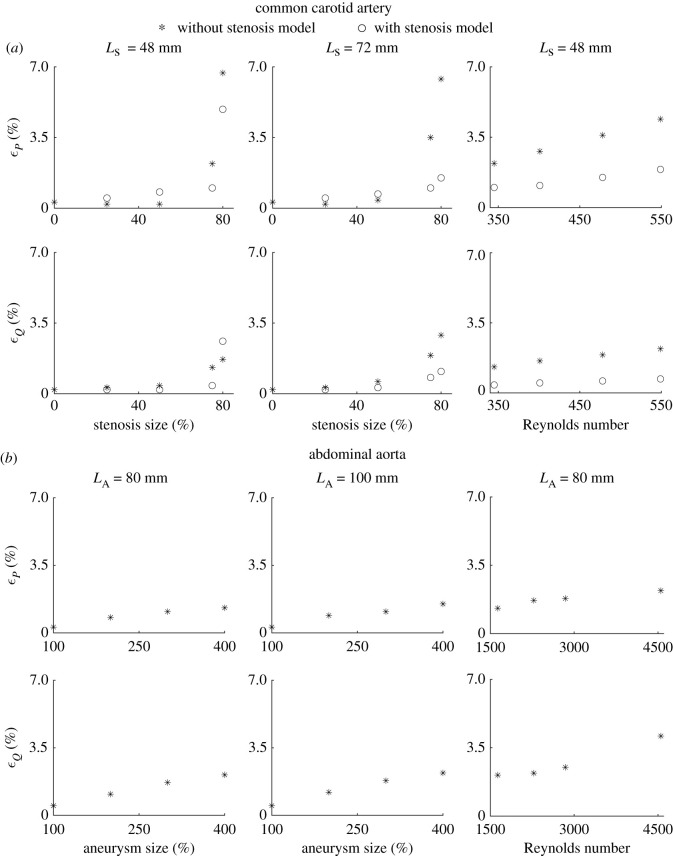
Relative RMSEs for the common carotid artery (*a*) and abdominal aorta (*b*). (*a*) Errors for blood pressure, *ε*_*P*_, and flow, *ε*_*Q*_, in the middle side with stenosis size, for a 48-mm-long stenosis (left) and a 72-mm-long stenosis (middle), and with Reynolds number for a 75%, 48-mm-long stenosis (right). Relative errors are provided for the 1-D model with (circles) and without (stars) the stenosis model. (*b*) *ε*_*P*_ and *ε*_*Q*_ in the middle side with aneurysm size, for 80-mm-long (left) and 100-mm-long (middle) aneurysms, and with Reynolds number for a 400%, 80-mm-long aneurysm (right).

Comparisons between 1-D and 3-D pulse waves for all cases simulated in this study (i.e. for different stenosis sizes and lengths, and *Re*) are provided in electronic supplementary material, figures S1–S5. Relative errors for both pressure and the flow increased with the increasing stenosis size, decreasing stenosis length and increasing *Re*. Moreover, discrepancies in both pressure and flow predictions occur mainly in systole, leading to greater systolic than diastolic relative errors, as well as maximum relative errors usually occurring within systole. Figure 5*a* shows *ε*^RMS^ at the middle of the stenosis for all cases simulated in this study. Overall, these were smaller when using the stenosis model, leading to *ε*^RMS^ smaller than 4.9% for pressure and 2.6% for the flow for all cases studied, with the greatest *ε*^RMS^ obtained for the case with an 80%, 48-mm-long stenosis and *Re* = 345.

The pressure drop across the stenosis, *δP*, increased with increasing stenosis size and length, and increasing *Re* (figure 4*c*). The relative error metric *ε*_*δP*_ used to quantify the accuracy of the 1-D model estimate of *δP* increased with increasing stenosis size and *Re* and decreasing stenosis length, with the maximum absolute error for *δP* being 9.8 mmHg (figure 4*c*, *Re* = 549). Furthermore, the stenosis model reduced *ε*_*δP*_ considerably: *ε*_*δP*_ ≤ 10.0% versus ≤ 4.7% for all cases studied.

#### 3.1.2. Abdominal aorta with an aneurysm

**Figure 6.**
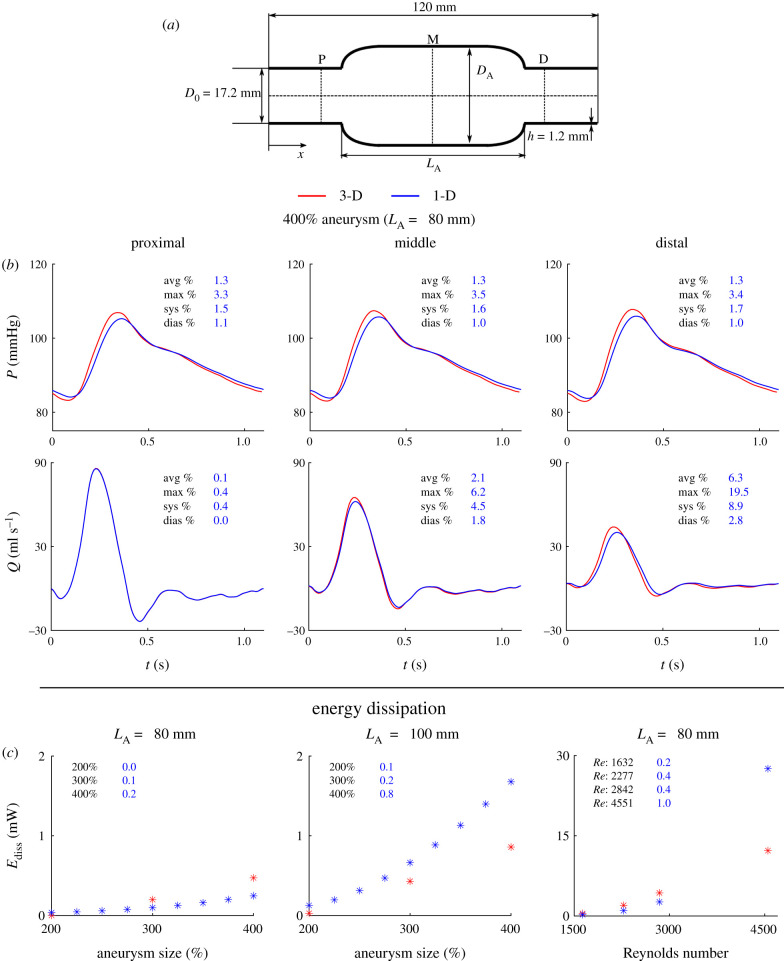
Results for the abdominal aorta with an aneurysm (1-D versus 3-D modelling). (*a*) The vascular geometry is characterized by the length of the aneurysm, *L*_A_, the initial, *D*_0_, and aneurysm, *D*_A_, diameters and the wall thickness, *h*. (*b*) Pressure and flow rate with time at the proximal (P), middle (M) and distal (D) sides shown in panel (a), calculated using the 3-D model (red) and 1-D model (blue) for a 400%, 80-mm-long aneurysm with a Reynolds number of 1632. Average (avg), maximum (max), systolic (sys) and diastolic (dias) relative error metrics are shown in each panel. (c) Energy dissipation, *E*_diss_, from the proximal to distal side with aneurysm size, for an 80-mm-long (left) and a 100-mm-long (middle) aneurysm, and with Reynolds number for a 400%, 80-mm-long aneurysm (right; note the change in the *y*-axis scale). Relative errors, $\epsilon_{E_{\mathrm{diss}}}$, calculated by equation (2.11) are shown in each panel for the cases in which 3-D model results were available.

Figure 6*b* shows 1-D model predictions for pressure and flow waves at 5 mm proximal, the middle and 5 mm distal to a 400%, 80-mm-long aneurysm, together with corresponding 3-D results. At those sites, *ε*^RMS^ remained smaller than 1.6% for pressure waves and 7.3% for flow waves when an AAA of up to 400% in size was present in the middle of the vessel, for a physiological *Re* of 1632. The 1-D model formulation was able to capture the main features of both pressure and flow waveforms produced by the 3-D formulation. Relative errors in blood pressure were smaller, and relative errors in blood flow were larger, than those obtained for the CCA with a stenosis. For all cases, 1-D modelling was able to reproduce the decrease in flow amplitude towards distal locations—which was more prominent with the increasing aneurysm size and length—while maintaining mean blood flow and pulse pressure constant along the blood vessel. In addition, the 1-D formulation was able to capture the decrease in pulse pressure along the vessel with increasing aneurysm size. 1-D model predictions systematically underestimated the amplitude of 3-D model pressure and flow waves, though relative errors in systolic and diastolic pressure were smaller than 2.2% and 1.1%, respectively, whereas relative errors in systolic and diastolic flow were smaller than 9.0% and 3.9%, respectively.

Electronic supplementary material, figures S6–S8 show all the 1-D versus 3-D comparisons for pressure and flow waveforms that were studied for the abdominal aorta case. All relative error metrics increased with increasing aneurysm size and length, and increasing *Re*. As in the CCA case, discrepancies in both pressure and flow predictions occurred mainly in systole rather than in diastole. Relative errors for pressure were similar throughout the vessel, but relative errors for the flow increased from inlet to outlet. For all the cases simulated in this study, *ε*^RMS^ in the middle of the aneurysm was less than 2.2% for pressure and 4.1% for flow, with the greatest values of *ε*^RMS^ corresponding to a 400%, 80-mm-long aneurysm with *Re* = 4551 (figure 5*b*). In addition, both the energy dissipation, *E*_diss_, and the relative error metric, $\epsilon_{E_{\mathrm{diss}}}$, used to quantify the accuracy of *E*_diss_ calculated by the 1-D model increased with increasing aneurysm size and length, and with increasing *Re*, with the maximum absolute error for *E*_diss_ being 15.3 mW (figure 6*c*, *Re* = 4551). $\epsilon_{E_{\mathrm{diss}}}$ values were 0.2% or less for an aneurysm length of 80 mm, and increased to 0.8% or less when the aneurysm length increased to 100 mm, and further increased to 1.0% or less when the *Re* increased from 1632 to 4551.

#### 3.1.3. Aortic bifurcation with a stenosis and an aneurysm

**Figure 7.**
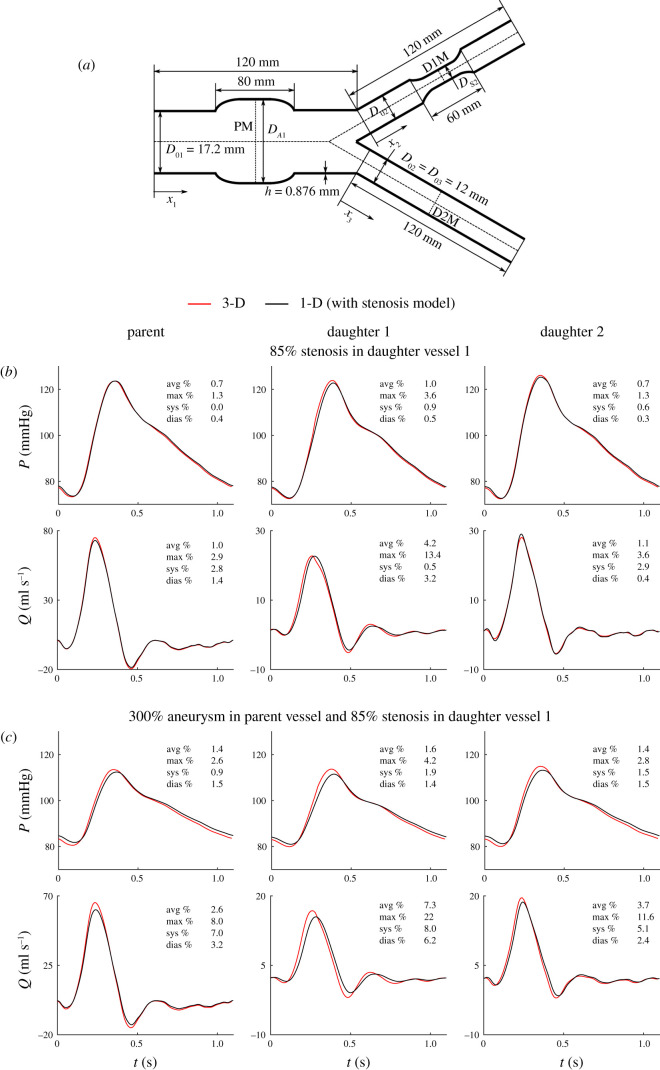
Results for the aortic bifurcation with an aneurysm in the parent vessel and a stenosis in daughter vessel 1 (1-D versus 3-D modelling). (*a*) The vascular geometry is characterized by the initial, *D*_01_, *D*_02_, *D*_03_, aneurysm, *D*_A1_, and stenosis, *D*_s2_, diameters and wall thickness, *h*. (*b*) Pressure and flow rate with time in the middle of the three vessels as indicated in panel (*a*), calculated using the 3-D model (red) and 1-D model (black) with an 85% stenosis in the middle of daughter vessel 1 (top) and a 300% aneurysm in the middle of the parent vessel together with an 85% stenosis in the middle of daughter vessel 1 (bottom). Average (avg), maximum (max), systolic (sys) and diastolic (dias) relative error metrics are shown in each panel.

The stenosis model was used in all 1-D simulations of the aortic bifurcation since, when this model was used, previous results had shown a considerable decrease in relative errors for both pulse wave morphology and pressure drop across the stenosis. The 1-D formulation with the stenosis model was able to reproduce the morphology of 3-D model pressure and flow waveforms accurately when adding an 85%, 60-mm-long stenosis to the left iliac artery of the aortic bifurcation model (figure 7*a*). *ε*^RMS^ for pressure and flow waves in the middle of the three vessels of the aortic bifurcation were, respectively, less than 1.0% and 4.2%. 1-D versus 3-D comparisons of pressure and flow waveforms are provided for the middle point of the three vessels (figure 7*b*) and at their inlets and outlets (electronic supplementary material, figure S10). Pressure *ε*^RMS^ values were similar to those obtained for the CCA with an 80%, 72-mm-long stenosis (2.2% or less; see electronic supplementary material, figure S4, bottom), and considerably smaller than the 80%, 48-mm-long stenosis case (5.4% or less; electronic supplementary material, figure S2, bottom). Flow *ε*^RMS^ values were slightly larger than those obtained for the CCA with an 80% stenosis, both 48 and 72 mm long (3% or less). Furthermore, 1-D modelling was able to simulate the pressure drop across the stenosis in the left iliac artery with a relative error of $\epsilon_{\delta P}=0.5\%$, which is smaller than the corresponding values obtained for the CCA with an 80%, 48-mm-long stenosis (2.8%) and an 80%, 72-mm-long stenosis (1.0%).

Adding a 300%, 80-mm-long AAA to the aortic bifurcation model with an 85%, 60-mm-long stenosis in the left iliac artery increased the *ε*^RMS^ value to 7.3% or less in the middle of the three vessels of the aortic bifurcation reported above for the flow wave (figure 7*c*). However, the 1-D formulation was still able to capture the decrease in pulse pressure in the three vessels observed in the 3-D results and maintain $\epsilon^{\mathrm{RMS}}\leq 2.0\%$ for pressure, despite increasing systolic and diastolic relative errors from 1.0% or less to 2.0% or less. 1-D versus 3-D pressure and flow waveforms at the inlets and outlets are shown in electronic supplementary material, figure S11. The increases in flow and pressure relative errors were caused by an underestimation of the amplitude of 3-D model pressure and flow waves, as observed for the abdominal aorta case with an AAA (figure 7*c*). Furthermore, *ε*_*δP*_ across the stenosis in the left iliac artery remained almost unchanged compared with the case without an AAA (0.8% versus 0.5%). The 1-D formulation was able to estimate the energy dissipation across the AAA with a relative error of $\epsilon_{E_{\mathrm{diss}}}=0.1\%$, which was smaller than the values obtained for the same size AAA in the abdominal aorta case.

For both cases, discrepancies in both pressure and flow predictions occurred mainly in systole rather than in diastole. Moreover, the greater relative errors for pressure waves were obtained in the middle of the stenosis and, for the second case, in the middle of the AAA as well. For example, *ε*^RMS^ increased up to 1.6% for pressure and 7.3% for the flow compared with the 0.7% or less and 1.7% or less errors, respectively, obtained for the baseline simulation (electronic supplementary material, figure S9).

### 3.2. Assessment against *in vitro* measurements

**Figure 8.**
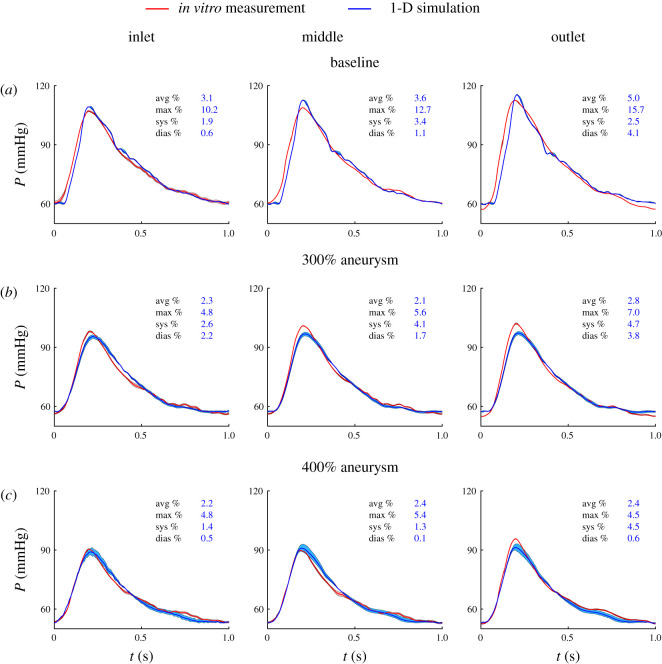
Results for the abdominal aorta with an aneurysm (1-D versus *in vitro* modelling). Pressure with time measured *in vitro* (red) and simulated by the 1-D model (blue) at the inlet, middle and outlet of the vessel for the baseline (*a*), 300% aneurysm (*b*) and 400% aneurysm (*c*) geometries. The plots also show the standard deviation (shaded areas) of the ensemble average of each *in vitro* and simulated pressure wave. Average (avg), maximum (max), systolic (sys) and diastolic (dias) relative error metrics are shown in each panel.

Young’s modulus measured on five specimens of the abdominal aorta phantoms by the electromechanical test system was 690 ± 23 kPa. Figure 8 shows the pressure measured *in vitro* and simulated by the 1-D model at the inlet, middle and outlet of the three phantoms using this Young’s modulus. The thick red and blue lines are, respectively, the ensemble-average pressure waveforms from the *in vitro* measurements and simulated using the 1-D model. For the *in vitro* measurements, the shaded areas indicate the ensemble standard deviation of the pressure wave. The shaded areas of the 1-D model pressures were obtained as follows. Ensemble-averaged inflow boundary condition and mean Young’s modulus were used to simulate the ensemble-averaged pressure wave; ensemble-averaged inflow ± standard deviation and mean Young’s modulus ± standard deviation were used to simulate the upper and lower pressure values of the shaded areas.

The 1-D formulation was able to reproduce the *in vitro* pressure waves with average, systolic and diastolic relative errors smaller than 5.0% for all three cases. These errors were larger than corresponding errors obtained by comparison against 3-D model pressures (2.0% or less). Unlike in the comparisons against 3-D model pressures, relative errors were not affected by the aneurysm size and increased with the distance from the inlet. 1-D modelling was able to capture the decrease in pulse pressure with increasing aneurysm size observed *in vitro*, which was also observed in the 3-D model pressures. Overall, *ε*^RMS^ values were similar along the three aortic phantom geometries, which is consistent with the results obtained when comparing against the 3-D model pressures.

## 4. Discussion

We have tested the accuracy of 1-D model pressure and flow waveforms in a series of idealized vascular geometries representing the abdominal aorta, common carotid and iliac arteries with different sizes of stenoses and/or aneurysms. These arteries were selected for being preferable locations for aneurysm or stenosis in large vessels [57,58]. This is the first attempt, to our knowledge, to test systematically and quantitatively the accuracy of the 1-D formulation in arteries with localized changes in luminal cross-sectional area due to the presence of vascular disease. Overall, we have shown that 1-D modelling is able to reproduce the main features of pressure and flow waves, pressure drop across the stenoses and energy dissipation across aneurysms observed in the 3-D models that were used as the ground truth. Under physiological Reynolds numbers (*Re*), relative RMSEs were smaller than 5.4% for pressure and 7.3% for the flow in the vessels studied, for stenosis and AAA sizes of up to 85% and 400%, respectively. The errors are slightly larger than corresponding relative errors previously reported for the aorta and other large arteries with normal vascular geometries, in which relative RMSEs smaller than 2.1% and 5.0%, respectively, were obtained when also using 3-D model data as the ground truth (table 1). We have also shown the ability of 1-D modelling to simulate pressure waves acquired from phantoms of the abdominal aorta, leading to relative RMSE values of 5.0% or less, which are comparable to those obtained in previous studies (10.0% or less) for which *in vitro* data were used as the ground truth. Our study provides additional support for the use of 1-D modelling to accurately simulate pressure and flow waves in large diseased arteries with a reasonable computational cost. Moreover, all the 1-D, 3-D and *in vitro* data from this study are available online, providing a comprehensive reference dataset to support the development of 1-D models and numerical schemes in diseased arterial vasculatures.

We first discuss the effect of a stenosis and/or aneurysm on 1-D model pressure and flow waves, and then the limitations and significance of our results.

### 4.1. Stenosis

The primary source of discrepancies between 1-D and 3-D model pulse waves across a stenosis was underestimation by 1-D modelling of the pressure loss across the stenosis. Using 3-D modelling, complex flow patterns and increased wall shear stresses can be observed around a stenosis region [59–62]. These complex flow phenomena cannot be described by the 1-D formulation, which led to relative errors in pressure and flow waves that increased with increasing stenosis size. Moreover, relative errors were considerably greater for pressure than for flow in the CCA case, especially at locations proximal to the stenosis. This is because the mean flow rate and, to a large extent, flow wave patterns were determined by the inflow boundary condition and preserved throughout the vessel according to the law of conservation of mass (equation (2.1)). Instead, the pressure gradient required to drive the prescribed inflow waveforms increased with the increasing pressure drop across the stenosis (this drop got larger with increasing stenosis size, length and *Re*), leading to greater pressures in proximal locations. Pressures in distal locations were determined by the outflow boundary condition, which was identical in both 1-D and 3-D formulations, leading to much smaller relative errors in pressure.

By introducing an empirically based stenosis model—accounting for the pressure loss across the stenosis—into the 1-D formulation of the CCA case with stenoses larger than 50% in size, relative errors were overall considerably reduced; for example, relative RMSE decreased from a maximum of 10.6% in pressure and 3.2% in flow to a maximum of 2.2% and 1.6%, respectively, in an 80%, 72-mm-long stenosis. Moreover, pressure drops across the stenosis, *δP*, were remarkably better reproduced: the maximum value of the relative error metric *ε*_*δP*_ used to quantify the accuracy of the 1-D model estimate of *δP* was at least halved and decreased by up to 90% in some cases.

Comparing the aortic bifurcation and CCA cases, relative errors for the pressure were considerably smaller in the aortic bifurcation case with an 85% stenosis in the iliac artery than in the CCA case with an 80% stenosis. This is in agreement with the increase in relative pressure errors with increasing Reynolds number (*Re*) observed in the CCA case. Indeed, under normal physiological *Re*, the iliac *Re* was smaller than the CCA *Re* (*Re* = 106 versus 345). Furthermore, relative errors in pressure were similar throughout the vessels of the aortic bifurcation case, since proximal pressures did not increase with the presence of the stenosis to drive the prescribed inflow. This is because volume flow rates could be redistributed towards the iliac artery without the stenosis, which provided less resistance to blood flow. In fact, the flow distribution between the iliac artery with and without stenosis,changed considerably, especially during systole (peak flow rates in the middle of the iliac arteries were 25 ml s^−1^ and 29 ml s^−1^, respectively). The systolic flow split in the two iliac arteries was slightly different in 1-D and 3-D modelling (46.2/53.8% in 1-D versus 45.1/54.9% in 3-D), leading to larger relative errors in 1-D model flows than in the CCA case.

In general, the largest differences between 1-D and 3-D model pressures and flows in the two cases with a stenosis occurred in systole, whereas the diastolic predictions were much closer, in agreement with previous results obtained in healthy vasculatures [11,19]. Systolic flow is fundamentally nonlinear and advection/inertia-dominated, and, therefore, larger differences between the two formulations were observed as expected. Conversely, the physics of blood flow becomes increasingly linear and inertia-free with increasing time in diastole, facilitating the task of the 1-D formulation to reproduce pressure and flow waves in diastole [63].

The empirically based stenosis model used in this study originated from the *in vivo* experiments in dogs by Young *et al.* [34] and *in vitro* measurements by Seeley & Young [35]. In those experiments a range of stenoses from 50% to over 90% in size, 6.5 mm to 101.4 mm in length, and *Re* from less than 100 to over 1000 were considered under pulsatile and steady flow conditions. We have shown that 1-D modelling with this stenosis model can provide pulse waveforms much closer to the 3-D model waveforms in stenosis sizes from 50% to up to 85%, stenosis lengths from 48 mm to 72 mm, and *Re* ≤ 550, which are within the corresponding ranges of values considered in the *in vivo* and *in vitro* experiments.

### 4.2. Aneurysm

The primary cause of relative errors in the aneurysm model was the inability of 1-D modelling to describe the intricate 3-D flow patterns inside the aneurysm, which can be observed in 3-D model simulations [9,64,65]. As a result, relative errors were considerably greater for the flow than for the pressure when an AAA was present, in both the abdominal aorta and aortic bifurcation cases. Unlike in the CCA case with a stenosis, the pressure gradient required to drive the prescribed inflow in the abdominal aorta case was not significantly affected by the presence of the AAA and, hence, relative errors in pressure stayed low and varied little along the vessel. On the other hand, relative errors in the flow increased towards distal locations and with increasing AAA size and length. Furthermore, flow waves were determined by the inflow boundary condition in locations proximal to the AAA, and were affected by the size and length of the AAA in distal locations. With increasing AAA size and length, more flow was stored in the AAA region in systole, resulting in the aneurysm region expanding and further increasing vascular compliance, which is directly proportional to the luminal cross-sectional area [50]. In the 3-D models, the external tissue support reduced the expansion of the aneurysm region, leading to smaller compliances and, hence, the Windkessel effect was greater in the 1-D simulations. As a result, pulse pressures and flow amplitudes were greater in the 3-D than in the 1-D simulations, with their differences increasing with increasing AAA size and length.

The interplay between the presence of a stenosis and an aneurysm was studied in the abdominal aortic bifurcation case. Adding an AAA to the aortic bifurcation model with an 85% iliac artery stenosis increased relative errors, especially in the flow. The presence of the aneurysm increased compliance in the vasculature, especially in the 1-D model that was without tissue support. The additional compliance in the abdominal aorta decreased the amplitude of the blood pressure and flow towards the outlet of the aorta. As a result, the pressure and flow amplitudes in the left iliac artery with a stenosis also decreased, leading to larger relative errors when both a stenosis and an aneurysm were present.

We have used the energy dissipation across the AAA (the difference in energy flux between a proximal and a distal location) to evaluate the ability of 1-D modelling to account for the effect on pressure and flow waves of the intricate 3-D flow patterns within the AAA. This dissipation metric was proposed in the study by Marsden *et al.* [56] and takes into account changes in both blood flow and pressure between several vascular locations. In general, our results have shown that 1-D modelling can provide both accurate pulse waveforms and energy dissipations for aneurysm sizes up to 400% and *Re* ≤ 4551.

The maximum relative RMSEs obtained when testing the 1-D formulation against the *in vitro* measurements were slightly larger than the RMSEs found when assessing 1-D modelling against 3-D modelling (5% or less versus 2.7% or less). This is in agreement with the results presented in previous studies comparing 1-D versus *in vitro* and 3-D data, as shown in table 1. In addition, the relative errors in pressure varied little with increasing size of the aneurysm, similar to the results found when comparing 1-D and 3-D simulations: the maximum difference in the relative RMSEs between a 300% and a 400% aneurysm was 0.4%. In our study, the measured and simulated pressure waves did not show the oscillations observed in the study performed by Sazonov *et al.* [38]. A possible reason for this is the greater Young’s modulus used in Sazonov *et al.*’s study, which was about four times larger than that used in this study, resulting in decreased compliance in the experimental vasculature; however, further investigations are required to confirm this. Another possible reason for the oscillation-free measurement results found in our study is the presence of water filling the gap between the phantom and the rigid experimental table, which prevented any resonance from occurring between the phantom and the rigid table.

### 4.3. Limitations

*In vivo* data were not used in this study as the ground truth. Instead, 3-D model and experimental data were used, which have the fundamental advantage of reducing the uncertainty of the 1-D model physical parameters. The 1-D and 3-D formulations shared identical boundary conditions and had equivalent vascular geometries and material properties. Relatively accurate measurements of the physical parameters used in the 1-D models of the abdominal aorta phantoms were possible for all parameters except for fluid density and viscosity. In this study, therefore, we have shown that the 1-D formulation is able to capture the main features of pressure and flow waves across stenoses/aneurysms in large vessels with reasonable accuracy, provided that accurate measurements of all physical parameters are used. As a result, this study provides a theoretical lower bound of relative errors to be expected when testing 1-D modelling against *in vivo* data.

We have investigated a particular numerical implementation of the 1-D model theory, which accounts for nonlinear effects and is able to provide physiological features of human pulse waveforms in normal vasculatures [66]. More complex 1-D formulations than the one considered here have been proposed, for example those accounting for wall visco-elasticity [13], space-varying and time-varying velocity profiles [16,67] and highly nonlinear terms [16,68]. However, assessing all these formulations was beyond the scope of this study. Furthermore, laminar flow was assumed in both 1-D and 3-D model simulations. This approach fits most of the simulation cases for which the Reynolds numbers (at peak flow velocity) were smaller than 2100. However, some extreme cases in which the Reynolds number went up to 4561 may benefit from using turbulent flow models.

The low relative errors obtained in this study are comparable to those obtained in other studies involving normal arterial geometries (e.g. those listed in table 1). They should, however, be confirmed in patient-specific geometries with anatomically correct stenoses and/or aneurysms and in arterial tree models with the larger systemic arteries simulated using 1-D modelling.

Lastly, the impact of neglecting energy losses at the aortic bifurcation has not been analysed, although it should be insignificant because of the small relative errors obtained in the aortic bifurcation case. This is in agreement with the findings of previous studies [11,12]. The impact of other geometrical features such as tapering, curvature and torsion has not been studied here, but it has been previously shown to also be insignificant and affecting mainly 3-D flow patterns rather than pulse wave morphologies [11].

### 4.4. Significance

This study was motivated by the scarceness of test cases for benchmarking 1-D numerical schemes in diseased vasculatures and our desire to provide an accessible reference dataset. Previous studies have used 1-D modelling to study pulse wave propagation across stenoses [69,70] and aneurysms [38], before having comprehensively verified 1-D modelling in such diseased vasculatures. Our results suggest that 1-D modelling offers a good balance between accuracy and computational cost in arteries with localized changes in luminal cross-sectional area, supporting the use of 1-D modelling in those previous studies on diseased vasculatures, and in future studies. We have shown that discrepancies in both pressure and flow predictions obtained by using 3-D and *in vitro* data as the ground truth are similar to those obtained in the studies shown in table 1 that also tested 1-D modelling in normal vascular geometries using 3-D model and *in vitro* reference data. Moreover, we have shown that the 1-D formulation is inexpensive to compute in diseased vasculatures compared with the 3-D formulation (minutes versus days).

Having a robust and fast 1-D formulation that is able to simulate arterial haemodynamics in diseased vasculatures will allow us to investigate indices and algorithms that can be obtained from pulse wave analysis and which may enable early detection of stenoses and AAA. In addition to existing indices, such as fractional flow reserve for coronary artery stenosis [71,72], new indices, such as the energy dissipation across an AAA proposed in this study, can be assessed using 1-D modelling under a wide range of cardiovascular conditions, which is currently prohibitive—from a computational standpoint—when using 3-D modelling. Moreover, our results support the use of 1-D modelling to create datasets of thousands of ‘virtual’ (computed) subjects with different sizes of large-artery stenoses and AAA for assessing the performance of such indices and algorithms, following our existing approach [73], which so far has been used to create healthy virtual subjects only. For instance, deep-learning algorithms for estimating the size of an AAA from a peripheral pressure wave can be developed using such datasets of virtual subjects [74].

## 5. Conclusion

We have shown the ability of the 1-D formulation—with an empirical model of energy losses across stenoses—to capture the main features of pressure and flow waveforms in the CCA with stenoses of up to 85% in size, the abdominal aorta with aneurysms of up to 400% in size and the abdominal aorta with an AAA of 300% in size and an iliac stenosis of 85% in size. This study provides additional support for the use of 1-D modelling to accurately simulate pressure and flow waves in large diseased arteries with a reasonable computational cost. All numerical and experimental data generated in this study are freely available and are a valuable resource for future research.

## Data accessibility

Additional data are included in the electronic supplementary material.
